# Numerical Investigation of Very Low Reynolds Cross Orifice Jet for Personalized Ventilation Applications in Aircraft Cabins

**DOI:** 10.3390/ijerph20010740

**Published:** 2022-12-31

**Authors:** Florin Ioan Bode, Ilinca Nastase

**Affiliations:** 1 Mechanical Engineering Department, Technical University of Cluj-Napoca, Muncii Boulevard Nr. 103-105, D03, 400114 Cluj-Napoca, Romania; 2 Building Services Department, CAMBI, Technical University of Civil Engineering in Bucharest, 66 Avenue Pache Protopopescu, 020396 Bucharest, Romania

**Keywords:** lobed jet, axis switching, numerical simulation, LES, LDV, PIV, personalized ventilation

## Abstract

This study focuses on the numerical analysis of a challenging issue involving the regulation of the human body’s microenvironment through personalized ventilation. We intended to first concentrate on the main flow, namely, the personalized ventilation jet, before connecting the many interacting components that are impacting this microenvironment (human body plume, personalized ventilation jet, and the human body itself as a solid obstacle). Using the laminar model and the large eddy simulation (LES) model, the flow field of a cross-shaped jet with very low Reynolds numbers is examined numerically. The related results are compared to data from laser doppler velocimetry (LDV) and particle image velocimetry (PIV) for a reference jet design. The major goal of this study is to evaluate the advantages and disadvantages of the CFD approach for simulating the key features of the cross-shaped orifice jet flow. It was discovered that the laminar model overestimated the global jet volumetric flow rate and the flow expansion. LES looks more suitable for the numerical prediction of such dynamic integral quantities. In light of the computational constraints, it quite accurately mimics the mean flow behavior in the first ten equivalent diameters from the orifice, where the mesh grid was extremely finely tuned. From the perspective of the intended application, the streamwise velocity distributions, streamwise velocity decay, and volumetric flow rate anticipated by the LES model are rather well reproduced.

## 1. Introduction

It has long been known that traveling by plane has long been associated with the spread of viruses through infected passengers and maybe through in-flight transmission [1]. In addition to dirty surfaces and intimate contact with other passengers, it is a problem with biology, physics, and ventilation. After a droplet expires, its liquid content immediately starts to evaporate. Some droplets, known as nuclei, become so small that air current transmission affects them more than gravitational pull, which would otherwise take their infectious content meters and tens of meters away from the point of origin [2]. Everyone produces very little water droplets (aerosols) (0.3–20 m) that are 97% water and 3% solutes when they breathe, speak, cough, or sneeze (salts, proteins, and other substances) [2,3]. It has been suggested that the relative humidity (RH) of the air is crucial for the spread of airborne illnesses [4,5].

The water in the droplets evaporates more quickly in low-RH settings (less than 40%), reducing the mass of the droplets, extending their duration on the surface of the water, and extending the distance they travel [6]. The chemical composition of the droplet changes as the level of salts increases and the droplet crystallizes in a solid state. In some instances, this will lead to the development of a floating microcapsule where bacteria and viruses will be kept for a longer period of time and have a larger chance of infecting others. On the other hand, the cabin atmosphere experiences very low relative humidity (RH) during flight conditions, and the air circulation encourages the movement and mixing of microdroplets that could be contaminated with viruses.

Space optimization and safety were priorities when the majority of current airplanes and their environmental control systems (ECSs) were created more than 30 years ago. The ECS will maintain the cabin’s temperature and pressure at roughly 24 °C and 0.8 bar, respectively. At the end of long-distance flights, the humidity can be as low as 2–3%. During cruise conditions, the supplied air temperature is roughly 18 °C with a relative humidity of 10–20% or less [7].

The air supply is typically located at the top of the cabin and the exhaust air is located at the bottom, with mixing air being circulated throughout the cabin [8]. The mixing system in the cabin will produce recirculation zones (large vortex flows), which will cause localized air stagnation. In addition to having a negative effect on cabin air quality, mixing ventilation does not encourage the exclusion of pollutants. An examination of the literature revealed that the air distribution currently used in airplane cabins is inadequate at preventing the spread of airborne infectious disease viruses [9]. A set of gaspers (orientable nozzles) above the passenger seats on airplanes provide a customized ventilation system that may be adjusted for flow rate and direction.

Mixing between the air that is given and the air that is already present in an enclosure is the primary idea behind mixing ventilation (MV), which is utilized in both buildings and aircraft nowadays. Because clean air is delivered through diffusers that are far from the occupants, it has time to mix with contaminated ambient air before being absorbed in the case of dangerous pollutants such as virus-laden aerosols. Although it is a recent advancement in the realm of building air distribution, personalized ventilation (PV) is employed extensively in the automotive sector [10,11,12]. The fundamental goal of PV is to improve comfort by bringing clean air close to each occupant’s face and granting them some control over their microclimate. On the other hand, PV systems could be employed as barriers to prevent cross-infection in crowded environments such as airplanes.

The idea behind protective personalized ventilation (PPV) in this situation would be to deliver clean air specifically to a person’s breathing zone while enabling some control over the flow rate and flow direction. The ASHRAE and REHVA position documents on airborne infectious diseases propose PV and other ventilation techniques (such as dilution ventilation, local exhaust, and source control ventilation) [13,14] as efficient controls and preventions for the spread of SARS-CoV-2. This suggested approach is in line with data showing that systems using both PV and MV offer greater protection against airborne infection than those that just employ MV.

When Pantelic et al. [15] examined the effectiveness of a desk-mounted PV diffuser, they found that it might shorten the exposure time to cough droplets as well as their peak aerosol concentration. Displacement ventilation DV systems are frequently employed in buildings and have been demonstrated to be more successful in eliminating contaminants than mechanical ventilation MV systems [16].

There have been several efforts to put forth fresh ideas based on the displacement notion to lessen the spread of viruses that cause airborne infectious diseases and/or enhance cabin air quality. In a mock-up of an A320 cabin, Schmidt [16] and Müller [17] compared MV with DV. Schmidt [16] found that a DV system could result in an uncomfortable feeling of overheating while an MV system had a larger chance of draft. In [18], Xu et al suggested a mechanism for distributing air beneath aisles with the aim of raising relative humidity from 10% to 20%. Zhang [19] also suggested a PV system with air terminals incorporated into the armrests of commercial airline seats.

It is necessary to exert some control over the breathing zone’s flow characteristics in order to improve the quality of the air there and enable PV flow to interact with the free convection flow of the human boundary layer. This boundary layer must be penetrated in order for fresh air to be delivered to the BZ via PV (i.e., at speeds greater than 0.3 m/s). Previous studies show that the convective boundary layer of the human body and the PV flow interact strongly [18].

Air terminal devices (ATDs) [20] or furniture-integrated air diffusers are two examples of PV designs that are normally positioned a particular distance away from the breathing zone of the passenger. In an attempt to enhance PV efficiency, efforts have been made to reduce the flow of the boundary layer by cooling the desk where the person is seated [21] or to dissipate it using forced ventilation [22]. As a result of the weakening of the convective boundary layer, the needed PV airflow was lowered from 8 L/s to 6 L/s, resulting in better levels of clean air for the occupant.

Reducing the distance that separates the passenger and the PV diffuser is another means to increase the performance of PV [23,24]. Due to this, the PV flow rates may be reduced to as low as 0.5 L/s, which results in the majority of the air that is breathed in (more than 90%) being clean air. Factors such as the discharging velocity, equivalent diameter of the diffuser, and shape of the diffuser all have a role in determining the outcome of this strategy [25].

An extensive experimental investigation was conducted by Xu et al. [19] to look into the flow interactions between the occupant’s thermal boundary layer and customized air. According to their research, the PV jet’s ability to penetrate the thermal plume may not be as challenging as first thought. They came to the conclusion that a reduced clean air penetration velocity may be used to conserve energy while keeping outside clean air levels the same. However, they also made the point that, depending on the location and direction of the non-uniform flow from the nozzle, particularly at a lower supply air velocity, the airflow disturbances in the breathing zone owing to the thermal plume still need careful attention. This was particularly accurate when the air supply was discharged at a lower velocity.

Lobed orifices were added to the perforated panel ceiling diffuser design for mixed ventilation, and it was discovered that they had improved initial spread due to greater induction, without affecting the jet throw length [20,21]. Large stream-wise structures were produced by the nozzle’s lobed edge, which are known to be the cause of the jet induction phenomena [20,21]. The work by Bolashikov et al. [22] looked at the possibilities of modifying the inserted jet’s properties by using a lobed nozzle design. By using the Personal Exposure Effectiveness index (PEE), developed by Melikov et al. [23], the quality of the air that was breathed was evaluated. Compared to a free circular jet or a plane jet, lobed geometry nozzles are said to have a broader initial spread but more mixing [23,26,27,28,29,30,31]. The initial side spread was thought to be advantageous since the inserted jet would better cover the mouth and the surrounds of the inhalation zone, resulting in increased PEE, for the close distances between the lobed nozzles and the face, according to the case in this article as well. However, the outcomes did not entirely support this hypothesis. The four-leafed lobed nozzle produced the lowest PEE. For distances of 0.04 m and 0.06 m from the mouth and a velocity of 0.4 m/s, the six-lobed jet performed as well as the circular and elliptical nozzles.

The inserted flow should not alter a person’s body’s thermal perception if air is dispersed close to their face [23]. If the initial velocity is high, there could be some local pain. Human test participants who participated in experiments to assess the performance of a rectangular diffuser built into an earpiece [32] with an initial velocity of around 1.7 m/s reported feeling uncomfortable. To lessen the unpleasant draft feelings, speeds have to be reduced by around three times (or 0.6 m/s) [33].

The air supply from a PV terminal unit is described as a jet (personalized flow) inserted in a transverse flow from the perspective of fluid mechanics (the upward free convective flow generated by the human body). The interplay between the PV jet and the free convective flow will determine how much clean, customized air is delivered to the inhalation zone [24,25]. The nature of the PV jet, the free convective flow, as well as the direction of the PV flow, all influence the interaction (i.e., from front, upward or downward, i.e., transverse, opposing or assisting the boundary layer flow). Characteristics of the free convective flow, such as strength and thickness, depend on a variety of variables, including the disparity between the surface body temperature and the air temperature in the room, the shape and posture of the body, the clothing worn, etc. The greatest velocity in the free convective flow at the face region can reach 0.3 m/s when the air temperature is between 20 and 24 °C [23]. The size, shape, and design of the air supply nozzle affect the properties of the customized flow [24,25].

The University of La Rochelle in France is credited with having been the first to propose the use of lobed geometries as a means of enhancing the passive control of air diffusion in buildings and vehicles. This line of research was later continued at the Technical University of Civil Engineering in Bucharest. In these two places, a great number of studies examining various lobed geometries in their various configurations have been carried out [20,21,26,27,28,29,30,31,32,33,34]. These studies demonstrated that the transverse shear induced solely by the lobed shape in the lobe troughs leads to a breakdown of the Kelvin–Helmholtz structures into “vortices segments” for specific conditions for heating, ventilating, and air conditioning (HVAC) systems, namely, low or moderate Reynolds numbers [21,26,31,33]. Our earlier research has given us some insight into the physical processes that gave rise to their particular performance. The streamwise structures that are continuously forming in the lobe troughs, at the consequent discontinuity zones, control the lobed jet self-induction [33]. In the case of the lobed nozzle [21], the lobe deflection angles increase the transverse shear and produce a strong vorticity field made up of large-scale structures that are closely associated to this shear, boosting the air mixing in comparison to a reference circular jet. The lobed nozzle’s near-field mixing improvement is also preserved as an entrainment advantage in the farther field.

The maximum streamwise velocities decrease exhibited comparable throws for the two flows despite the resulting gain in air induction seen for the lobed perforated panel flow [20]. For each elementary cross-shaped orifice studied, the air induction in the near jet field is controlled by the large scale structures developed in the orifice troughs [33]. This lobed orifice—i.e., cross-shaped—was found to be in our previous investigations [29] the most advantageous in terms of induction among a series of lobed orifice geometries (i.e., daisy orifice with six lobes, tear-shape lobed orifice with four lobes, rectangular lobed orifice with six lobes, and others). Additionally, it was discovered that when a cross-shaped impinging lobed jet was compared to a circular impinging jet, the wall shear rates and mass transfer in the impingement region were up to 175% and 40% greater, respectively [35].

In order to increase the performance of PV systems, it may be a good idea to build PV terminal units with lobed orifices, particularly cross-shaped orifices. Given the unique geometry of the lobed orifice, a lobed jet would, in fact, be able to cover a wider area around the mouth than a circular jet for the same effective exit area and exit volumetric flow rate. Additionally, it should be feasible to regulate the maximum velocity in the first and intermediate jet zones due to the lobed jet’s intensifying vortex dynamics, which should prevent discomfort from draughts in PV applications. The lobed diffuser must be optimized in relation to all the previously mentioned factors that affect a PV system’s efficiency. In the case of a parametric study, a numerical approach is more appropriate because experimental investigations take time. Additionally, we discovered in earlier research that the cross-shaped jet flow’s fluid dynamics are extremely complex due to the numerous physical processes and time scales involved [33]. In this situation, at very low Reynolds numbers, it is crucial to directly assess the flow behavior in a non-intrusive manner because any disturbance could have an impact on how the jet develops later. An answer to this problem is provided by computational fluid dynamics (CFD) reinforced by experimental validations using contemporary research methods such as laser doppler velocimetry (LDV) or particle image velocimetry (PIV) [36,37].

This paper presents a comparison between two numerical approaches aiming to analyze the capabilities of a commercial CFD code to reproduce the behavior of a low, transitional Reynolds number, cross-shaped orifice jet. As an experimental validation we used our previous experimental results from [33] for which the Reynolds number of the cross-shaped jet is found in a critic region. Indeed, a classification of the jet flow regimes [38] shows that for Reynolds numbers larger than 2000 the flow becomes very quickly turbulent, in the proximity of its exit plane. In this case, the well-known laws of expansion and entrainment, generally found in the literature are valid. For Reynolds numbers between 500 and 2000 (the range of PV interest), the jet flow is laminar in its initial region and then becomes turbulent, with a non-typical behavior which is changing with the level of Reynolds number in this range [39].

Unsteady numerical simulations of the flow field were made using the commercial CFD code Fluent, included in the Ansys package. Low velocities required the use of the incompressible solver. Numerous viscous models are included in Fluent, ranging from the most basic laminar model [40] through large eddy simulation (LES), RANS models, and hybrid LES-RANS models. Because a direct full simulation (direct numerical simulation—DNS) of the problem at hand is still beyond our processing capacity at this moment, LES provides a good compromise over RANS models, which are inadequate due to the flow’s extremely low Reynolds number.

Furthermore, in our situation, jet flow instabilities are not reproduced by RANS models. Therefore, we decided to use the viscous LES model for the numerical simulation. Additionally, we wanted also to check the capabilities of the laminar model proposed by Fluent, since a laminar model would be less expensive in terms of computational resources. However, it is questionable whether such a model can reproduce the jet flow dynamics given the previously noted atypical regime.

## 2. Methods

### 2.1. Numerical Study—Investigated Geometry and Computational Details

**Figure 1:**
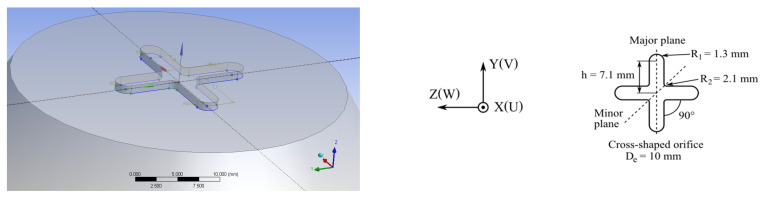
Investigated cross-shaped orifice.

**Figure 2:**
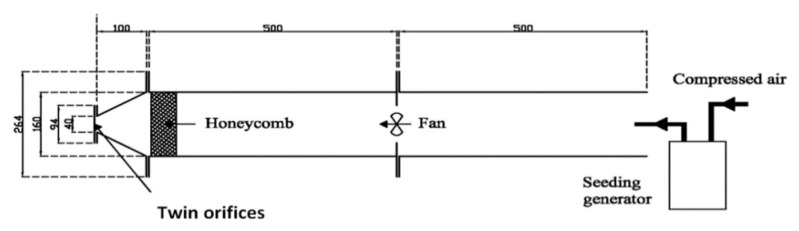
Experimental facility for air flow generation [29].

The air jet studied in this paper is generated using a cross-shaped orifice (Figure 1). The equivalent diameter based on the free area of the orifice is D_e_ = 10 mm. The numerical geometrical model was defined to reproduce the terminal part of an experimental facility described in Figure 2. The orifice has the same equivalent diameter as in the experimental study presented in [29,33], which is D_e_ = 10 mm. This experimental device was composed of an axial fan placed inside a one-meter-long metallic pipe of 0.16 m in diameter (see Figure 2). The level of turbulence at the jet exit was reduced thanks to a convergent duct that was installed at the pipe’s end. The orifice’s plate, built up from a 1 mm-thick aluminum sheet, is placed on the extremity of this convergent (Figure 2 [29]).

**Figure 3:**
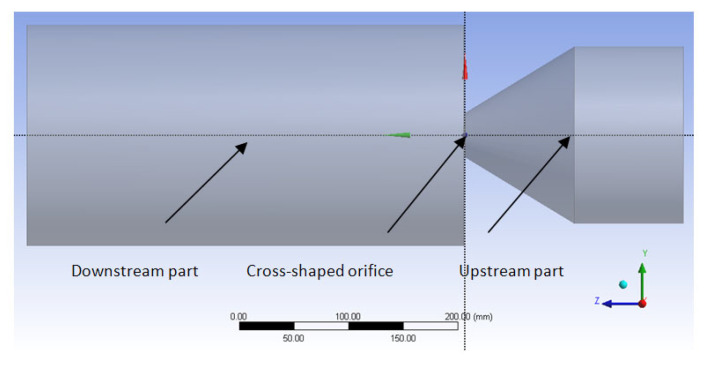
Investigated interest domain.

The geometrical model reproduced both the convergent and the orifice with its physical thickness, i.e., e = 1 mm (Figure 1 and Figure 3). Since the numerical simulation will be utilized to replace the very expensive experimental campaigns, the inlet condition must be supplied up-stream the orifice in order to optimize the diffuser’s design. We imposed a velocity magnitude of 0.0036 m/s, normal to the inlet boundary, and a turbulence intensity of 2% at the inlet plane of the domain’s upstream region. The inlet flow rate is equal to the experimental value of 7.57 × 10^−5^ m^3^/s. Based on the equivalent diameter D_e_ and the mean exit velocity U0mean (0.92 m/s) of the orifice, the initial Reynolds number is 515. The targeted PV application determines the selection of these exit conditions.

**Figure 4:**
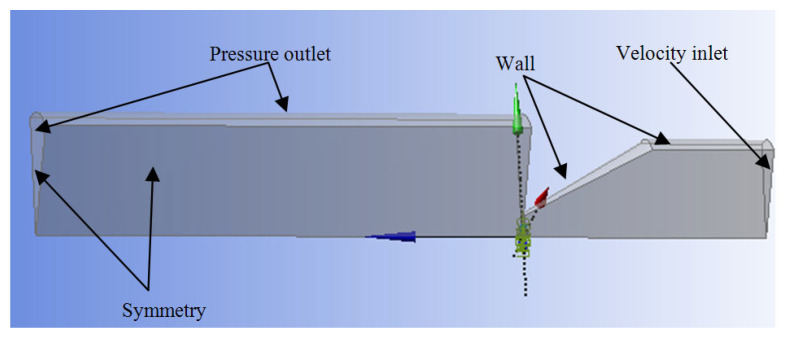
Geometry used for numerical simulation and boundary conditions.

**Figure 5:**
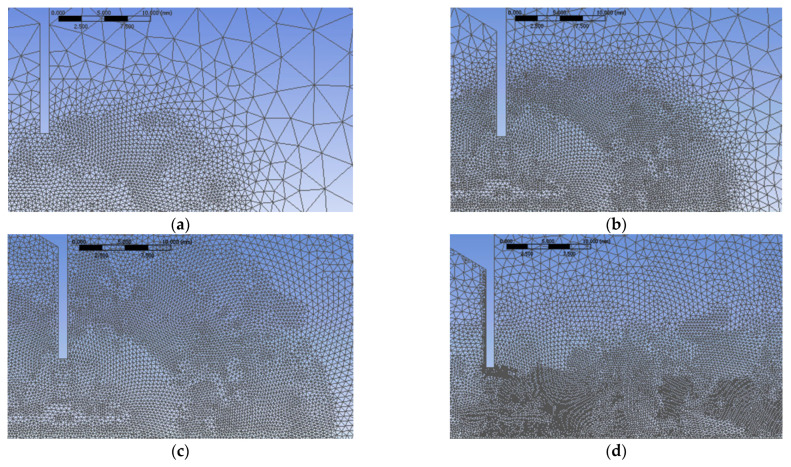
Detail of the mesh for the studied cases in the near exit orifice region: (**a**) 0.4 million elements, (**b**) 1.35 million elements, (**c**) 2.2 million elements, (**d**) 4.07 million elements.

The computational domain is composed of two parts (Figure 3) separated by the cross-shaped orifice plate. The upstream part of the domain reproduces the experimental convergent and the up-flow tube. The total length of the tube and convergent is 20 D_e_. The downstream part of the computed domain represents a cylinder with 20 D_e_ in diameter and 40 D_e_ in length. Only one eighth of the flow is modeled due to the symmetry of the problem (Figure 4). Figure 4 details the other boundary criteria. To determine the ideal mesh density for our situation without jeopardizing the limited computer resource or adding significantly to the computational cost, a mesh dependence research must be conducted [41]. In this study, a grid independence study was carried out using four different grid sizes of 0.4, 1.35, 2.2 and 4.07 million tetrahedral elements. As shown in Figure 5, where we detail the grids in the streamwise main plane and near to the orifice exit, all the utilized grids are extremely refined in the orifice plane. The results of this mesh dependence study are reported in more detail in [42].

For all the numerical grids, the smallest cells were located in the orifice thickness. The grid dependency study showed that there are no major differences between the 2.2 million elements grid compared with the 4.07 million elements grid, therefore the 2.2-million-element numerical grid was preferred for the numerical results comparison. For the 2.2-million-element grid, the dimension of the smallest cell in the orifice thickness was 0.05 mm, and the dimension of the largest cell was 0.15 mm. This way the thickness of the orifice was divided into 30 cells. The values of y^+^ in the orifice’s thickness were less than 0.1. For the other numerical grids, the maximum y^+^ value was 2.6 for the 0.4 million elements, 0.13 for the 1.35 million elements and 0.06 for the 4.07 million elements. On the outlet plane, the smallest grid cell was 0.5 mm thick, and the largest one was 3 mm thick.

Large structures that are present in the flow up to the grid limit are resolved using the LES approach. Because the greatest scales in the turbulent spectrum, in the boundary layer, are geometrically very small and require both a very detailed grid and a very small time step, LES often demands a very high resolution for wall boundary layers [43]. In contrast to Reynolds-averaged Navier–Stokes (RANS), LES requires that turbulence be resolved in both the wall parallel plane and the wall normal direction. Only very low Reynolds number flows and very small geometric scales allow for this. Only flows where there is no requirement for resolving the wall boundary layers or flows where the boundary layers are laminar due to the low Reynolds number are advised for the use of LES [44]. Our case meets these criteria, which led to the choice of the LES model to study the flow.

The numerical simulations were made using a segregated solver formulation. The difference between this formulation and coupled solver formulation is that the equations are solved sequentially instead of simultaneously. Using a control-volume-based method, Fluent transforms the differential governing equations into linear algebraic equations that can be solved numerically. For interpolation, a second-order upwind method was employed.

ANSYS FLUENT provides four distinct models for subgrid-scale turbulent viscosity modeling: Smagorinsky–Lilly, dynamic Smagorinsky–Lilly, WALE, and dynamic kinetic energy subgrid-scale model. Because LES only resolves the most energetic scales of the flow, modeling the smallest scales of turbulence introduces an uncertainty in the numerical results. The Smagorinsky–Lilly model [45] has several difficulties in resolving the subgrid-scale turbulent viscosity in more complex conditions, such as near a solid surface or in the presence of strong gradients.

An improvement was made by Germano et al. [46] and subsequently by Lilly [47] in the dynamic Smagorinsky–Lilly model. They developed a technique in which the Smagorinsky model constant, depending on the data provided by the resolved scales of motion, is dynamically derived.

Both dynamic Smagorinsky and Smagorinsky–Lilly models are generally algebraic models where subgrid-scale stresses are parameterized using resolved velocity scales. The assumption is that there is a local balance between the energy transferred through the grid filter scale and the small-scale kinetic energy dissipation by the subgrid. The subgrid-scale turbulence was better modelled by solving the subgrid-scale turbulence kinetic energy through the model proposed by Kim and Menon [48]. They proposed the dynamic kinetic energy subgrid-scale model which overcomes some of the limitations of the Smagorinsky–Lilly and dynamic Smagorinsky models. The dynamic kinetic energy subgrid-scale model solves an additional transport equation for the subgrid scale kinetic energy. The model predicts accurately the wall behaviour and transition and allows for energy backscatter; it also allows for a history of the kinetic energy. It is more computationally expensive as it solves an additional equation, and it performs explicit filtering.

For wall-bounded flows, the wall-adapting local eddy-viscosity (WALE) model [49] is intended to provide the proper wall asymptotic behavior. In contrast to the Smagorinsky–Lilly model, which generates non-zero turbulent viscosity, the WALE model creates zero turbulent viscosity for laminar shear flows. In these circumstances, the Wale model results in an accurate handling of the laminar zones in the domain. The WALE model retains the simplicity and low computational cost of the Smagorinsky model. Wall damping effects are accounted for without using the damping function explicitly. It predicts accurately the flow near the walls and transition.

In [50], the LES WALE model was used successfully in order to obtain similar transient flow field characteristics as in for the airflow experimental measurements inside a Boeing 737-200 cabin mock-up. In [51], the study assesses the performance of various turbulence models for transitional flows in an enclosed environment. They found that the discrepancies among the three LES models used were very small, with two of these models being LES WALE and LES dynamic kinetic energy subgrid-scale. In [52], the authors performed a comparison between RANS and LES models on a confined flow in an empty airplane cabin. Their conclusions were that LES models clearly and substantially outperform RANS models and the differences between the numerical simulations results using LES WALE and LES dynamic kinetic energy subgrid-scale are very small. The same conclusion emerged from [53], where it was shown that for an impinging jet, all LES models are capable of capturing small-scale dynamics, and WALE and dynamic kinetic energy subgrid-scale model models performed well in complex flow regions.

Therefore, in situations similar to the one of the present studies, the WALE model was preferred both to the Smagorinsky–Lilly model because of the better performance and to the dynamic kinetic energy subgrid-scale model because LES WALE is less computationally expensive.

Therefore, in our case we first started a steady numerical simulation with the laminar model enabled. After the solution has reached convergence in steady state, we started large eddy simulation (LES), using the WALE SubGrid Scale (SGS).

Due to the very low velocities, the flow is considered incompressible, and the pressure-based solver was used. Pressure-velocity coupling was made by the use of the SIMPLE algorithm. We selected a second-order, bounded central differencing scheme for the convective discretization schemes for all transport equations because it is the best option for LES because of its low numerical diffusion [44]. It was decided to use a second-order implicit approach for the transient formulation.

It is not necessary to achieve any convergence criterion to find a suitable value for the time step because the Fluent formulation is fully implicit [54]. Setting the time step at least one order of magnitude less than the shortest time constant for the scenario being modeled is one step that must be taken to appropriately simulate transitory occurrences. Fluent should ideally need between 5 and 10 iterations to converge at each time step. If Fluent needs more iterations for solution convergence, then it should be included in the previous interval, and then the time step should be reduced, or in the case when Fluent needs only a few iterations per time step, then the time step should be increased. Most of the time, a time-dependent problem has a very fast “startup” transient that degenerates rapidly. Considering these things, it is wise to choose a small-time step for the first 5 to 10 time steps. The time step will be increased as the calculation proceeds [43].

Additionally, we also ran numerical simulations for comparison with the Laminar model proposed by ANSYS FLUENT [44].

### 2.2. Experimental Validation of the Numerical Study—Measurement Techniques

The laser doppler anemometry measurements were performed by Nastase in [29]. Mean velocity fields measurements employed a two-dimensional system with two solid lasers: one Nd: Yag of 25 mW providing a monochromatic green beam (532 nm) and one Sapphire of 22 mW providing a monochromatic blue beam (488 nm). The sampling time during measurements was in the range of 60 to 100 s and the mean data rate ranged between 500 Hz to 4.8 kHz depending on the flow velocity at the measurement point.

The uncertainty of the measurement was estimated to be in the range of ±0.1 to ±2% for the U and V mean components near the exit plane, and in the range of ±1 to ±10% for the corresponding RMS velocities. For the V mean component and its corresponding RMS value, the uncertainty of the measurement was found to be even larger than 40% for axial distances larger than X = 2 D_e_. This estimation is based on the evaluation of a global bias error, depending on all the parameters susceptible to bias the measurements [55,56,57,58,59,60], and of a statistical uncertainty related to the data scattering around the mean values [59,61,62]. The probe was mounted on a three-dimensional traverse system with the ranges on the X, Y, and Z axis, as 690 mm, 2020 mm and 690 mm, respectively, and with the movement resolution and reproducibility of 6.25 µm. LDV data was acquired on a grid in the (YZ) plane at each X location. The grid spacing varied from 0.25 mm to 0.5 mm with the streamwise distance X.

For the vortex dynamics analysis of the studied jet, we confronted the numerical results with our earlier results obtained through the combination of classical PIV measurements and high-speed visualizations [33]. The PIV measurements are employed to acquire the instantaneous spatial distribution of the in-plane velocity in the transverse planes at the initial regions of the jet. Classical PIV measurements used a system composed of a CCD camera of 10^6^ pixels and a Nd: YAG laser of 120 mJ energy and 532 nm wavelength. The acquisition frequency of the PIV system was 15 Hz. The images calibration gave a spatial resolution of 26.8 µm per pixel which corresponds to a 42.8 × 31.7 mm^2^ field of view.

The PIV measurements were also performed by Nastase in [29]. We considered in each plane a number of 805 pairs of images which had been processed through an adaptive correlation algorithm with a multi-grid approach using window distortion and sub-pixel displacement of the windows. The validation condition was related to a threshold of 1.1 of the signals-to-noise ratio of the correlation. Less than 2% of the vectors were found to be non-valid and they were corrected using bilinear interpolation. The final grid was composed of interrogation windows of 32 × 32 pixels with 50% overlapping, which led to a spatial resolution of 1 mm and a vector spacing of 37.39 pixels. Generally, the particle displacement histograms had a bimodal distribution with no peak locking phenomenon. The visualizations were performed using a CMOS camera and a 4 W laser providing a 795 nm laser sheet. The maximum acquisition frequency of the visualization system is 5000 Hz for a 512 × 512-pixels window. For the visualizations, the air jet flows were seeded using incense.

For both LDV and PIV measurements, the air jet flow was seeded with paraffin oil droplets, 1…3 µm in diameter, using a liquid seeding generator.

**Figure 6:**
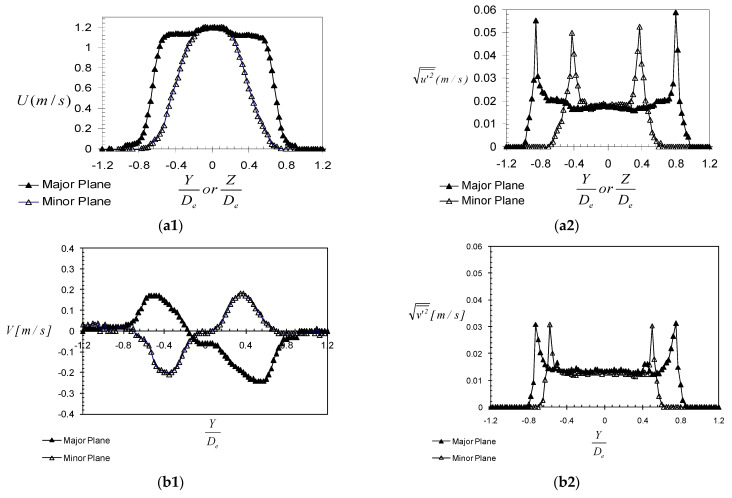

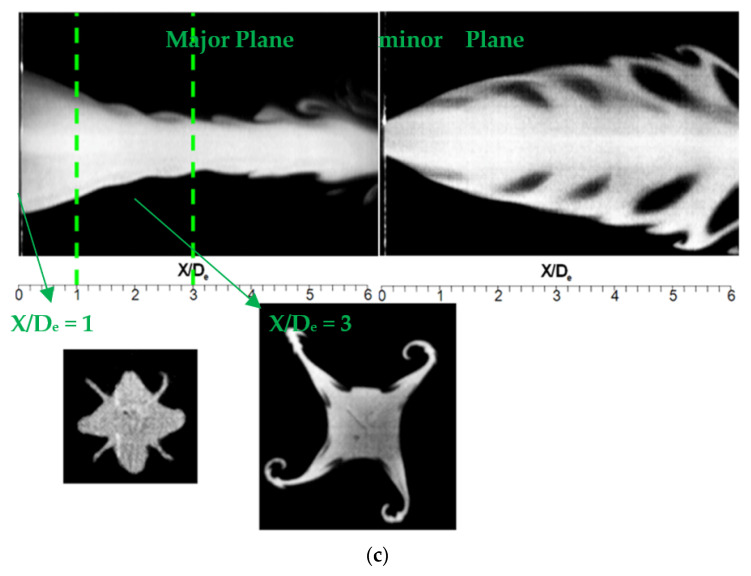
Exit velocity profiles in the experimental case: (**a1**,**a2**) streamwise component, (**b1**,**b2**) vertical component, (**a1**,**b1**) mean, (**a2**,**b2**) r.m.s; (**c**) high-speed visualization images of the cross-shaped jet (measurements performed by Nastase in [29]).

The exit profiles of the streamwise and vertical velocity components, from LDV measurements, at an axial distance X = 1 D_e_, are presented in Figure 6a,b. As displayed by the exit profiles of the streamwise velocity (Figure 6(a1)), a switching of the jet’s axes is present close to the exit. Indeed, in the minor plane of the jet, the streamwise velocity profile is almost twice as large as the one on its major plane. The switching over is visible on the visualization images of the streamwise Major and Minor Planes (Figure 6c). This phenomenon is generally associated with particular dynamics which may appear in the asymmetric jet flows [63,64,65,66].

## 3. Results and discussion

### 3.1. Global Analysis of the Flow and of the Tested Models

**Figure 7:**
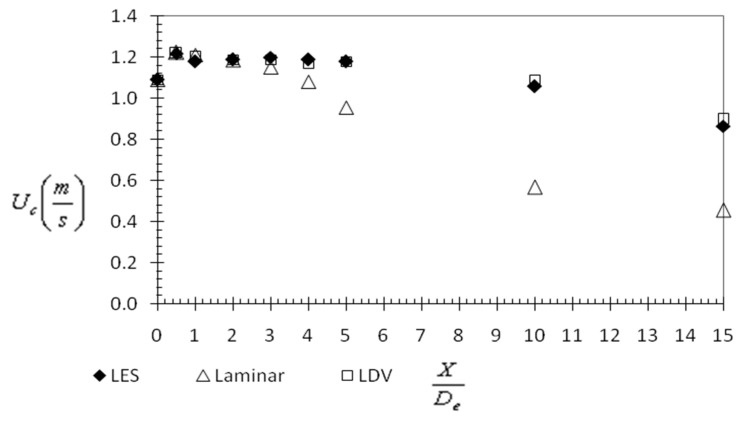
Evolution of axial velocity values in the axis of the jet.

As explained earlier, our research directions are oriented towards personalized ventilation applications. In this case, two global quantities are of main importance for designing an optimal air jet behavior: the streamwise velocity decay and the volumetric flow rate [67,68]. The first one gives information about how fast the velocity of the jet flow will attain values that are acceptable from the point of view of the draft sensation of the user, and the second one quantifies the ambient air induction. We represented in Figure 7 the evolutions of the streamwise velocity and of the volumetric flow rate for the experimental data, the LES and laminar models.

*Q* the volumetric flow rate was calculated by: Q=2π∫rU dr. Because we used a fixed criterion for determining the jet boundaries, we did not integrate all the measurement points at an axial location.

As our particular application is directly interested to quantify the mixing between jets generated by HVAC terminal units and their ambiance, we considered the 0.1 m/s criterion, for both the experimental and the numerical case, which define the extinction of the flow from the point of view of the thermal and draft comfort of the occupants [69].

In both figures, it is clearly visible that LES provides the best results. The laminar model tends to overestimate both the velocity decay and the entrainment rate. Due to the vena contracta effect of the jet (this phenomenon is commonly encountered in orifice jets [70]), the maximum value of centerline velocity near the jet exit is about 20% higher than bulk-mean exit velocity. Such a phenomenon is well predicted by the models.

**Figure 8:**
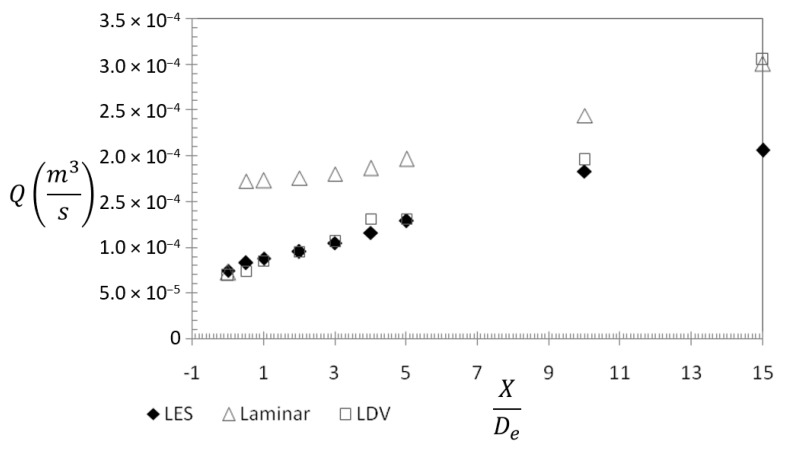
Streamwise evolution of the entrainment rate.

The high dissipation of the laminar model manifests as an over-prediction of the increasing rate of jet volumetric flow (Figure 8) and of the decay of the jet’s axial velocity (Figure 7).

**Figure 9:**
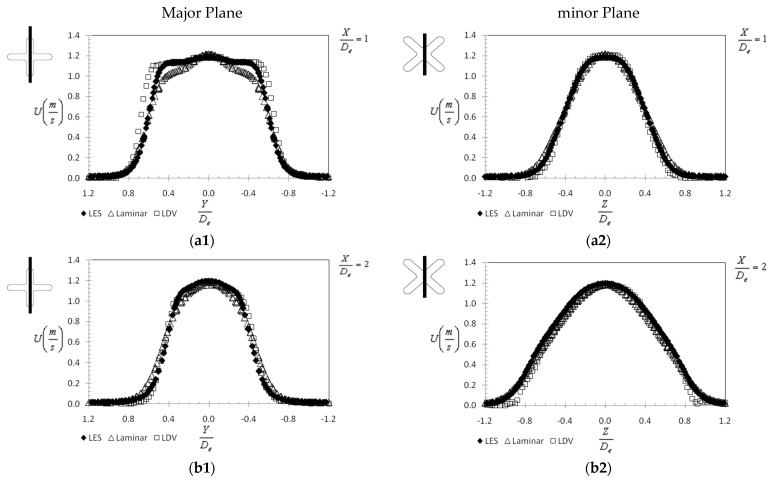

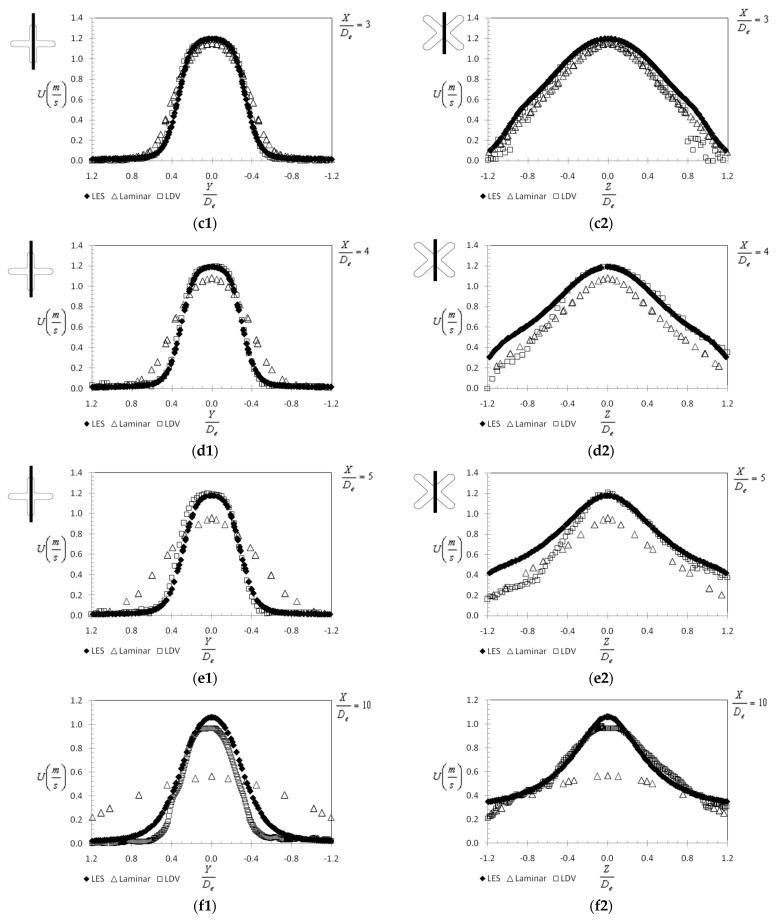
Mean axial velocity profiles at different distances from the exit plane (measurements performed by Nastase in [29]), (**a1**) Major Plane at X = 1 D_e_, (**a2**) minor Plane at X = 1 D_e_, (**b1**) Major Plane at X = 2 D_e_, (**b2**) minor Plane at X = 2 D_e_, (**c1**). Major Plane at X = 3 D_e_, (**c2**) minor Plane at X = 3 D_e_, (**d1**) Major Plane at X = 4 D_e_, (**d2**) minor Plane at X = 4 D_e_, (**e1**) Major Plane at X = 5 D_e_, (**e2**) minor Plane at X = 5 D_e_, (**f1**) Major Plane at X = 10 D_e_, (**f2**) minor Plane at X = 10 D_e_.

The expansion of the jet flow is also very important for the PV application since the use of a lobed jet instead of a classical circular jet aims to cover a larger area around the mouth. We represented in Figure 9 the mean streamwise velocity profiles of the jet flow at different axial distances (X = 1 D_e_, 2 D_e_, 3 D_e_, 4 D_e_, 5 D_e_ and 10 D_e_) from the exit plane, in the major plane (Figure 9(a1–f1)) and in the minor plane (Figure 9(a2–f2)), respectively. The expected axis switching phenomenon [31,33] is well reproduced by both models and is visible as a contraction of the jet flow on the major plane profiles with increasing axial distance, accompanied by an expansion of the flow on the minor plane profiles. Once again, the similarity (with experiments) of the results obtained through numerical simulation for the LES model should be noted, up to X = 5 D_e_. At X = 10 D_e_, the LES profile is slightly larger, and the axial velocity is greater compared to the measured profile. As for the laminar model, we can see a greater dissipation of the flow in comparison to reality beginning with X = 4 D_e_.

**Figure 10:**
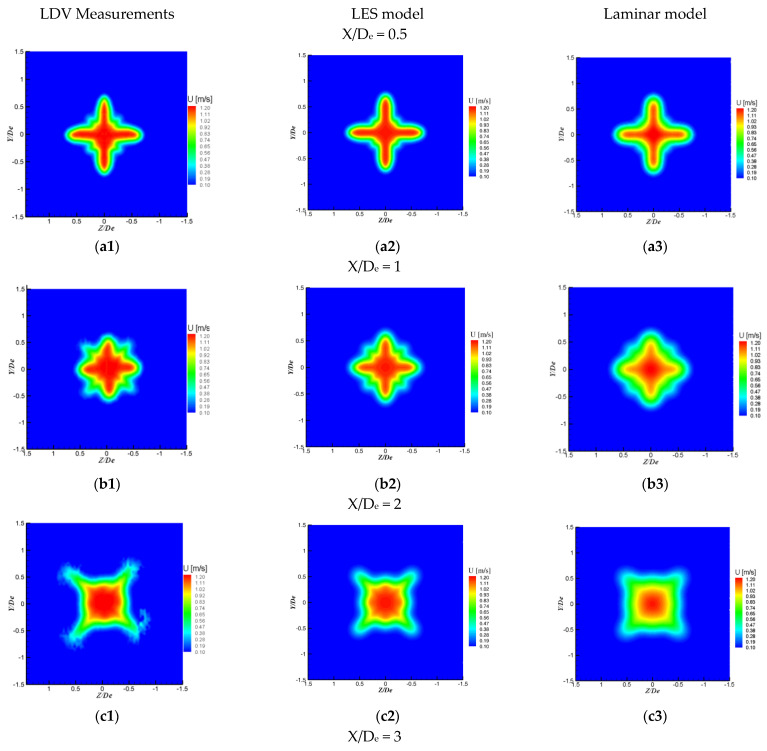

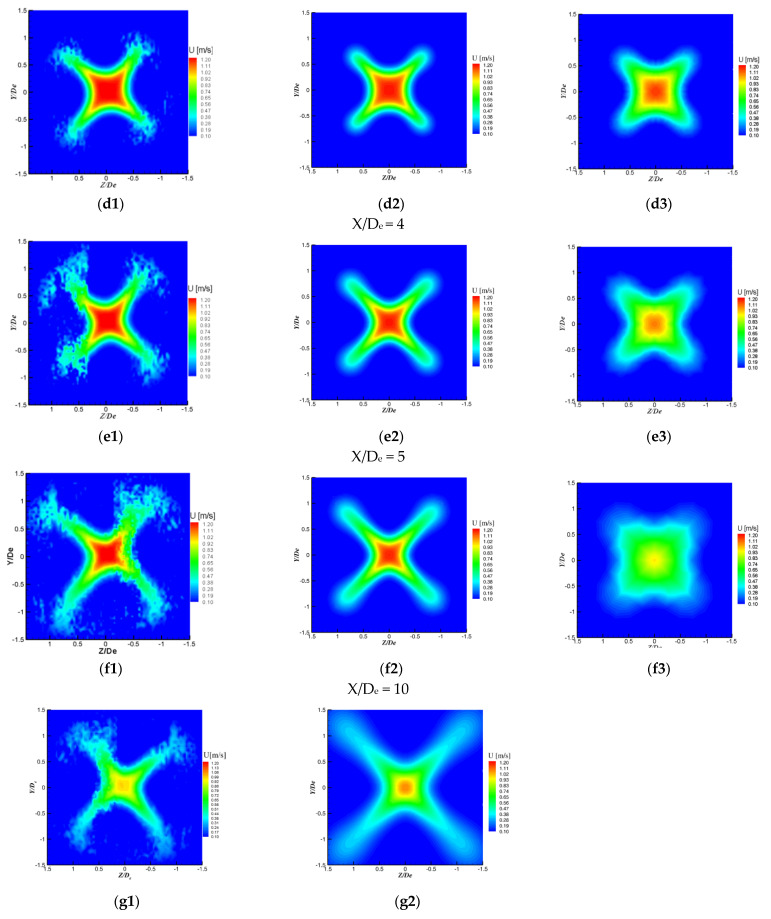

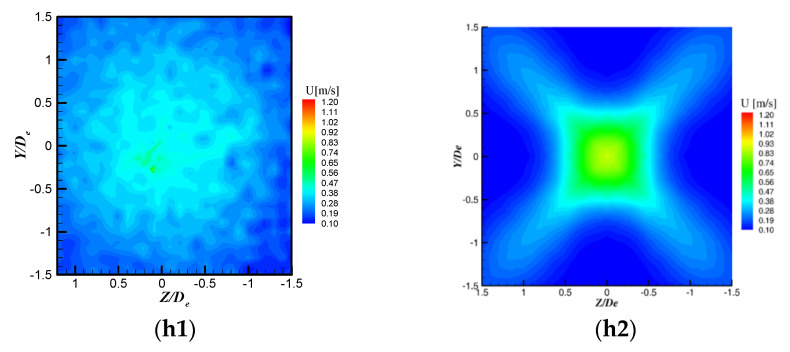
Streamwise velocity contours at different axial distances [29].

In the minor plane (Figure 9(a2–f2)), at X = 5 D_e_ a slight dissymmetry of the left side of the experimental profile makes us believe at first sight in a non-accordance between LDV and LES results. This is caused by the extremely unstable nature of the cross-shaped jet flow, which displays a slight “rotation” at this axial distance as visible in Figure 10(f1). At X = 10 D_e_ the LES profile is similar to the experimental profile, except for the central region where slightly larger values are observed.

The previous analysis based on the streamwise velocity profiles could be extended in Figure 10, where we represented the streamwise velocity contours at different streamwise positions for the two jets. Very close to the jet exit plane, the three contour plots corresponding to the LDV measurements, and respectively to the LES and laminar models, are quite similar. However, if we take a closer look at these contour plots, slight differences are revealed. The shape of the jet’s lobes is steeper on the figure corresponding to the experimental data (Figure 10(a1)), while for the numerical models the jet’s lobes are more rounded. The LES model better reproduced than the laminar model the shape of the small instabilities formed in the orifice’s troughs that will later develop into streamwise structures [33]. The laminar model also displays lower maximum velocity values. Furthermore, at X = 1 D_e_ (Figure 10(b1,b2)), the LES contours clearly reproduce the cross-shaped dynamics better than the laminar ones. The jet behavior predicted by the LES model is very good up to X = 5 D_e_. While LES and experimental results for the axial velocity decay and the volumetric flow rate evolutions agree very well up to X = 15 D_e_, the experimental data are not very well reproduced by the numerical streamwise velocity contours at X = 10 D_e_ and X = 15 D_e_. Indeed, in Figure 10(g1,g2), the central zone corresponding to the potential core is more restrained for the LES data compared to the experimental data but the periphery of the jet is more extended in the LES case. While at X = 15 D_e_, the experimental data indicate a total dilution of the jet flow, which no longer conserves its lobed shape, and the LES streamwise velocity contours still preserve the cross shape.

This way, the good prediction of certain dynamic quantities by the numerical model does not represent a guarantee for the good prediction of all dynamical characteristics of a strongly three-dimensional flow such as a lobed jet. Consequently, we have to avoid in the future concluding hastily about the performance of a model through a partial experimental validation. As a good prediction of the cross-section’s shape of the jet and of its expansion is important for the aimed application up to about X = 15 D_e_, a supplementary refining of the mesh downstream to X = 5 D_e_ is necessary. This will be possible in the very near future through the reinforcement of our computational means.

**Figure 11:**
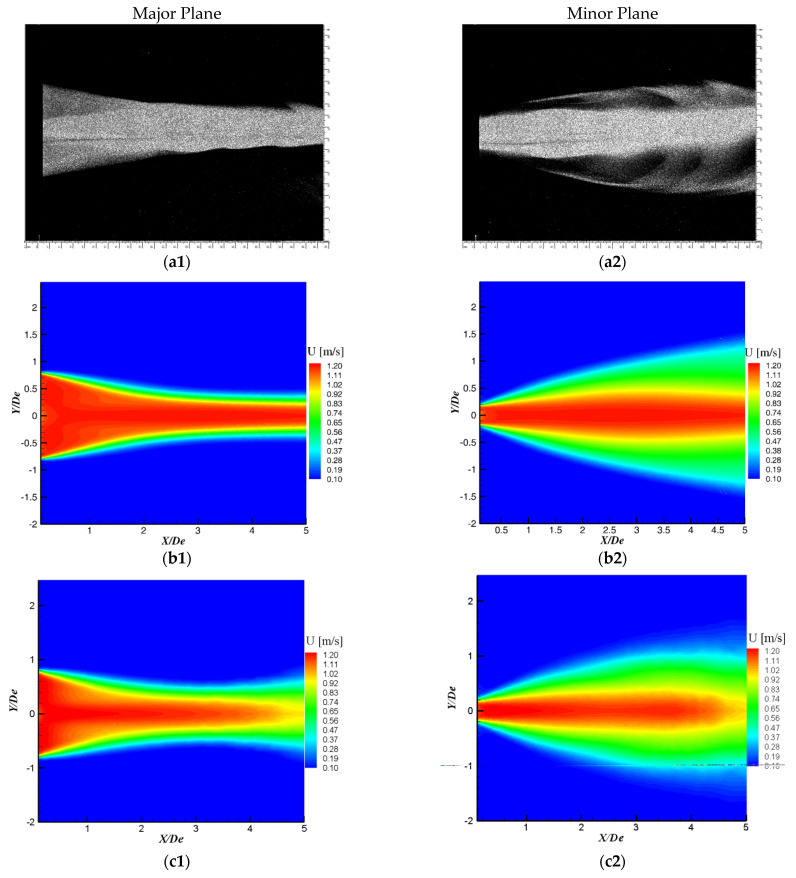
(**a1**,**a2**) Visualization images in the streamwise planes of the cross-shaped orifice jet; numerical streamwise velocity contours: (**b1**,**b2**) LES, (**c1**,**c2**) Laminar.

We wanted to analyze to the streamwise velocity contours in the major and minor planes as well, but we do not have the LDV or PIV measurements for the streamwise planes of the jet flow. In Figure 11 we compare, from a qualitative point of view, visualization images for the cross-shaped jet major and minor planes with contours of the streamwise velocity from the numerical simulations. On the images, the shapes of the central zones, where the tracer smoke is more condensed, correspond best with the potential regions from the LES streamwise velocity contours. For the laminar model, in the major plane, an expansion of the flow appears in the last part of the analyzed computational domain. This expansion corresponds to the premature deformation of the transverse profile of the flow towards a circular shape observable in Figure 10.

**Figure 12:**
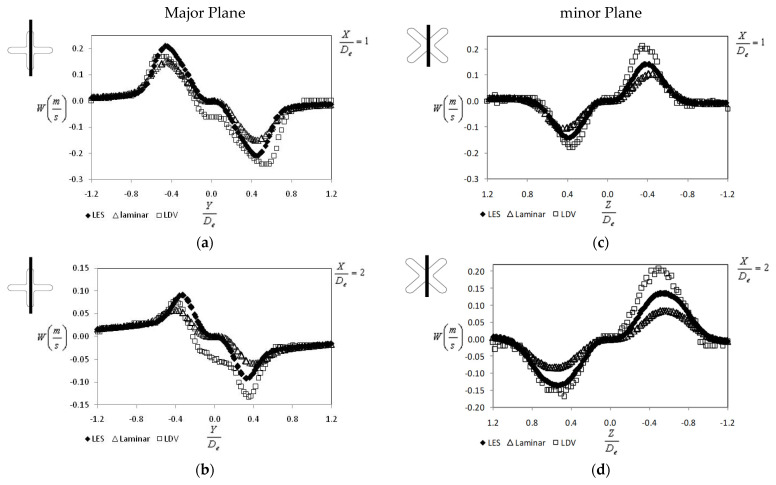
Mean vertical and lateral velocities profiles at different distances from the exit plane (measurements performed by Nastase in [29]) (**a**) in Major plane at X = 1 D_e_, (**b**) in minor Plane at X = 1 D_e_, (**c**) in Major plane at X = 2 D_e_, (**d**) in minor Plane at X = 2 D_e_.

In Figure 12 are presented the mean vertical and lateral velocity profiles for the first two diameters from the jet exit plane (X = 1 D_e_ and 2 D_e_). Given the low velocity values, the measurement uncertainty for the vertical and lateral velocity components are quite large (on the order of 10% close to the exit plane and even higher than 40% further downstream). Thus, we choose not to represent the corresponding profiles for other distances. Once again, for both major and minor planes, the LES model is closer to the experimental data than the laminar model.

At this point, it is clear to us that the laminar model is not appropriate for representing our lobed orifice jet in the chosen flow regime. As we will discuss further some aspects related to flow dynamics, we will consider in the next paragraphs only the LES numerical results.

### 3.2. Discussion of the Flow Dynamics and the LES Model’s Limits in the Studied Case

**Figure 13:**
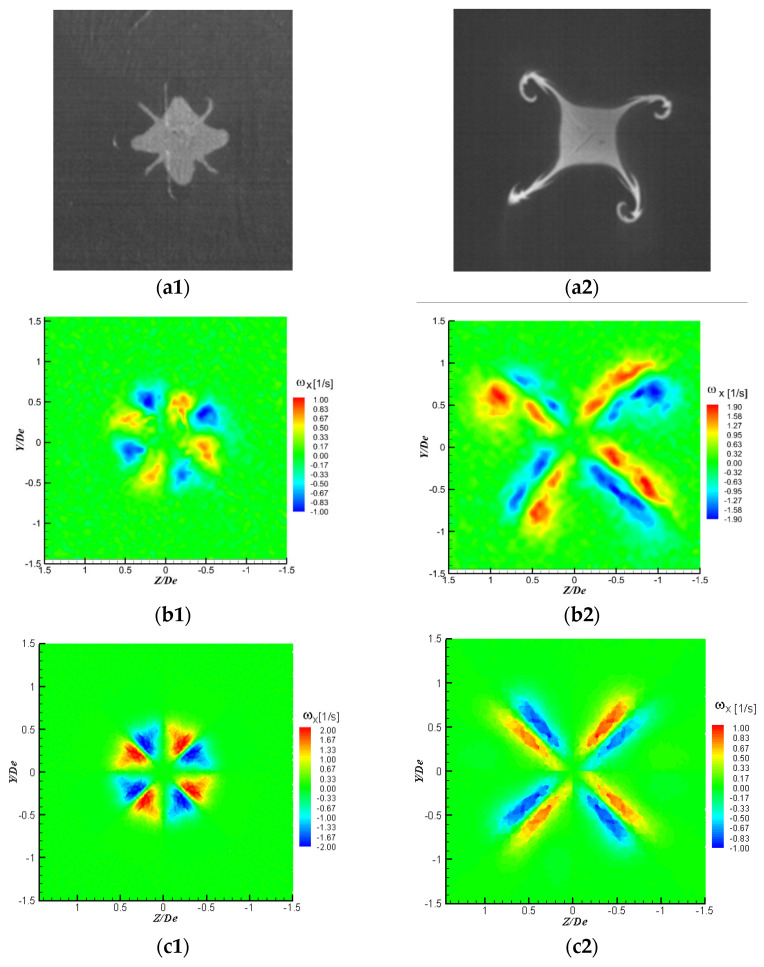
(**a1**,**a2**) Visualizations of the transverse plane of the jet, (**b1**,**b2**) mean vorticity fields from PIV measurements, (**c1**,**c2**) mean vorticity fields from LES; (**a1**,**b1**,**c1**) X = 1 D_e,_ (**a2**,**b2**,**c2**) X = 3 D_e_.

In Figure 13 are presented visualizations of the transverse plane of the jet as well as the fields of the mean streamwise vorticity component $\omega_{X}$ fields at X = 1 D_e_ and X = 3 D_e_, from PIV measurements and LES simulations. The streamwise vorticity component $\omega_{X}$ was defined as $\omega_{X}=\frac{D_{e}}{U_{0c}}\left(\frac{\partial W}{\partial Y}-\frac{\partial Z}{\partial X}\right)$. As could be observed in Figure 13, while at the first axial location the LES model tends to overestimate the maximum vorticity values, at X = 3 D_e_ it underestimates these maximum values. Nevertheless, the allure of the streamwise vorticity fields (Figure 13(c1,c2)) obtained by LES corresponds to the one of the real vorticity fields (Figure 13(b1,b2)).

The disposal and organization of the streamwise vorticity structures agree with the streamwise vortices visible on the visualization images at the same axial locations (Figure 13(a1,a2)). Indeed, streamwise vorticity organization can be seen as four regions of counter-rotating outflow vortice pairs from the jet center in the diagonal direction (Figure 13(b1,b2,c1,c2)). These are due to the blockage effect of the orifice troughs, similar to the one generated by tabs introduced in the trailing edge of a rectangular nozzle [65,66,71,72]; the flow impinges on the downstream face of the orifice plate and thus the cross-section of the jet core is bifurcated into four fingers observed in the flow visualization (Figure 13(a1,a2)). These structures eject the fluid from the jet core in the trough regions, outwards, along the radial directions, and at the same time entrain ambient air, inwards, at the lobe regions. This organization of the vorticity is well predicted by LES as well as the axis-switching phenomenon, which is also well predicted by the model.

**Figure 14:**
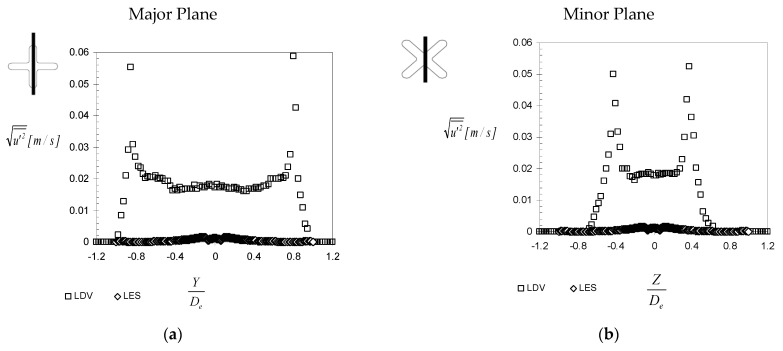
R.M.S of the streamwise velocity component profiles at the exit plane (measurements performed by Nastase in [29]) (**a**) Major Plane, (**b**) minor Plane.

As the LES model gives access to the temporal flow dynamics, we wanted to compare the r.m.s. of the velocity components profiles from both experimental and numerical approaches. In Figure 14 are represented the r.m.s streamwise velocity profiles for both major and minor planes at X = 1 D_e_. The values obtained from the LES model are smaller with four orders of magnitude. As this result was not expected, we modified the value of the initial turbulence imposed at the inlet of the computational domain from 2% to 5% and then, respectively, to 10%. No change could be observed on the r.m.s velocity profile behavior. We believe that this issue of the LES model is related to the very low Reynolds number of the studied flow and to a problem of reproducing the transition between the pipe flow and the free orifice jet.

We could impose, as some other authors have [73,74], the inlet conditions on the jet’s exit plane and use the experimental velocity and turbulence profiles. However, in the context of the PV terminal unit optimization, this approach is not suitable. We have therefore to accept this deficiency of the LES model in predicting the exit turbulence profile. Nevertheless, this bad prediction of the turbulence levels at the exit plane does not affect the mean flow prediction in its initial region.

**Figure 15:**
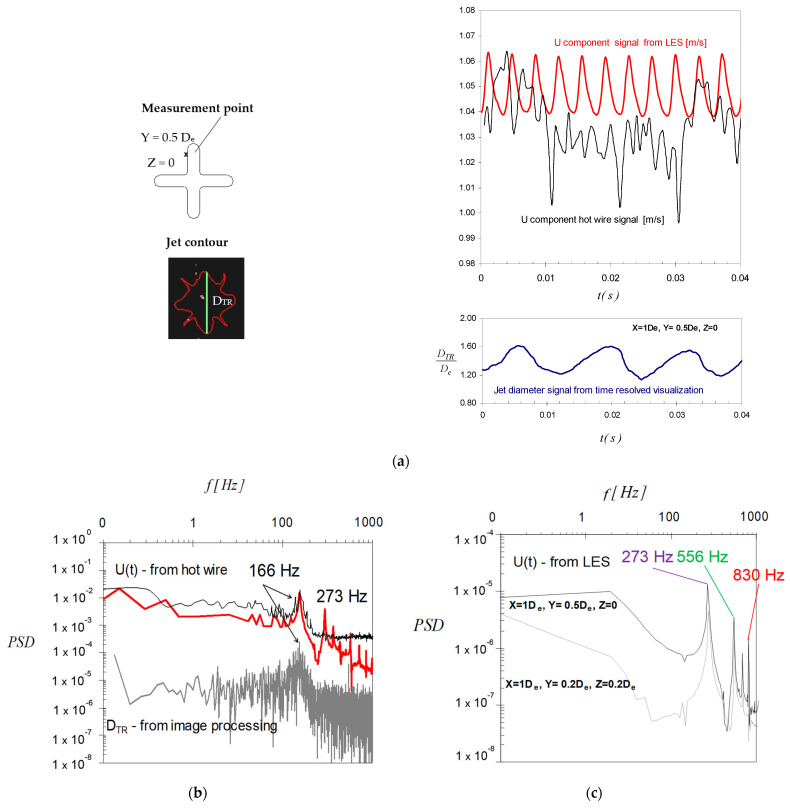
(**a**) Examples of velocity signals from a hot wire probe and LES simulation and of the jet flow dimension from time resolved visualizations (visualizations performed in [29]); (**b**) experimental spectra of the velocity signal—black line—filtered, red line—not filtered, and of the jet’s dimension signal—grey line; (**c**) spectra of velocity signals from LES.

In order to elucidate this “mystery”, we decided to take a look at the instantaneous variation in the velocity signal from hot wire measurements and LES. In Figure 15a are given examples of temporal variations of the streamwise velocity signals from a hot-wire probe [33] and from LES simulations at the measurement location defined by X = 1 D_e_, Y = 0.5 D_e_, Z = 0. For these signals, we found mean standard deviations of 13% for the hot wire signal and of 0.8% for the numerical signal. In Figure 15a is also represented the variation of the jet flow dimension D_TR_ extracted from time resolved visualizations using a procedure explained in detail in previous works [29,31,33,75]. It is interesting to observe the smooth variation of the jet’s dimension, which has the same sinusoidal appearance as the velocity variation from LES, in opposition to the noisy signal from the hotwire. However, the two signals have different periods. On the hotwire signal, two different periodical phenomena are visible. In Figure 15b,c are presented experimental spectra of the velocity signal and of the jet’s dimension—grey line and spectra of velocity signals from LES.

Both the filtered spectrum of the hot wire signal (black line) and the spectrum of the jet’s dimension signal (grey line) in Figure 15b revealed the same characteristic frequency of the Kelvin–Helmholtz vortices in our previous studies [29,31,33,75]. Anyway, if we take a look at the non-filtered spectrum from the hotwire, additional peaks are visible along with the fundamental frequency, the first one being located at 273 Hz. The characteristic frequency of 166 Hz is not captured by the LES model and the spectra in Figure 15c display a characteristic frequency of 273 Hz. This interesting phenomenon might have different explanations, but further investigations are necessary. Indeed, as could be observed in Figure 15c, the third peak on the same spectrum is at 830 Hz, which represents the triple of 166 Hz, which is the fundamental frequency from the experimental case.

### 3.3. Discussion of the Vortex Generation Mechanisms in Lobed Orifice Jets

**Figure 16:**
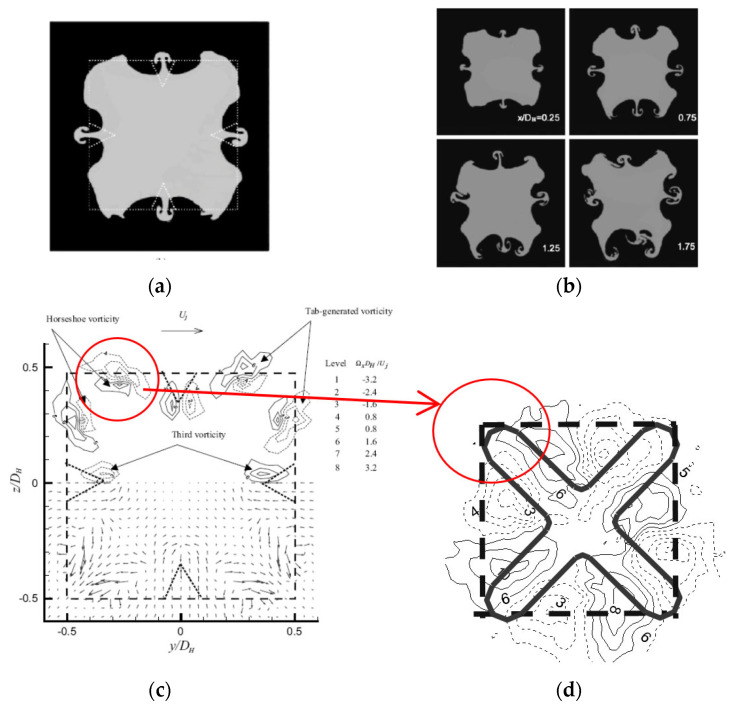
(**a**,**b**) Laser-induced fluorescence visualization of a jet flow from a square orifice with tabs [71], (**c**) transverse velocity and streamwise vorticity fields at the exit plane of the same jet flow from LDV measurements [71], (**d**) mean vorticity field at the exit plane of the cross-shaped jet from PIV measurements (measurements performed by Nastase in [29]).

As we mentioned earlier, in the previous studies [26,31,33] in which we investigated lobed plate orifice jets, we found that the lobed orifice geometry introduces a transverse shear in the lobe troughs following the similar effect of tabs introduced in the trailing edge of a rectangular nozzle [65,66,71,72], leading to a breakdown of the Kelvin–Helmholtz structures into “ring segments”. The lobed jet self-induction is controlled by the continuous development in the lobe troughs of the streamwise structures, at the resulting discontinuity regions. The same type of transverse flow dynamics were observed for a square jet with delta tabs [71], where the pressure hill formed upstream of the tab produces a pair of counter-rotating vortices [65,66,71] (see Figure 13 and Figure 16).

**Figure 17:**
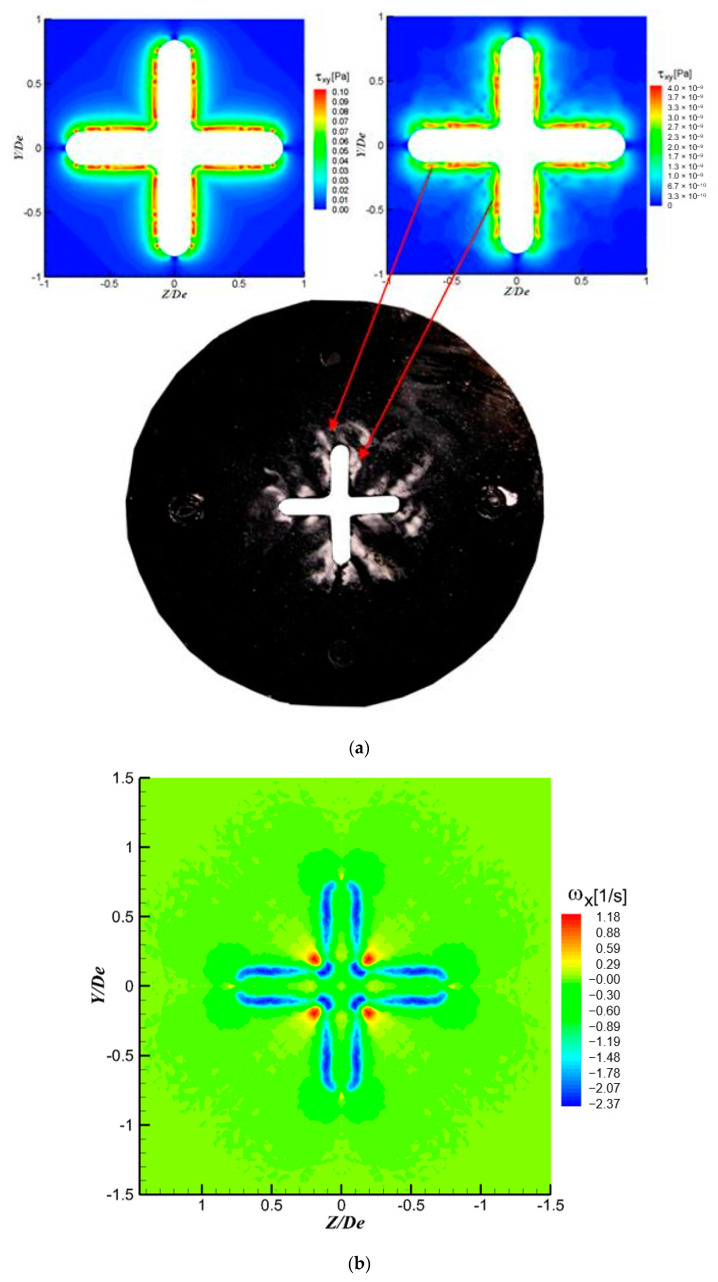
(**a**) Wall shear stress on the orifice’s plate surface inside the convergent from the LES, (**b**) streamwise vorticity distribution at X = 0.1 D_e_.

One of the advantages of using numerical methods in studying jet flow dynamics is given by the possibility of investigating regions that are not available normally through experimental approaches. As we were already questioning the phenomena upstream of our lobed nozzles and orifices, we performed surface visualizations in several cases [32,33], among which for the cross shaped orifice. A typical image of such a surface visualization is given in Figure 17a together with wall shear stress fields on the surface of the orifice plate, upstream of the exit plane, from the numerical LES model. The wall shear stress was defined by: $\tau_{YZ}=\mu \left(\frac{\partial W}{\partial Y}+\frac{\partial V}{\partial Z}\right)$.

Of course, this comparison has to be considered only from a qualitative point of view, but still, one could observe the similarities between the stagnation regions from the surface visualization and the shear stress distribution. Moreover, it could be deduced that the discontinuities of the Kelvin–Helmholtz structures and the appearance of the streamwise vortices is related to these distributions of the wall shear stress upstream the exit plane as suggested by the streamwise vorticity distribution very close to the exit plane (X = 0.1 D_e_). It should be noted that planes very close to the diffuser are not possible to observe experimentally through PIV or LDV due to the laser scattering by solid obstacles.

## 4. Conclusions

In the current context of energy-reducing consumption politics, new ways of controlling the micro-environment around the human occupants in buildings are being searched for. An innovative research direction aims to introduce high-performance lobed nozzles and orifices in personalized ventilation systems. The current study represents a first step from a larger experimental and numerical campaign for the optimization of a lobed orifice as a terminal unit for a personalized ventilation system. In this article, the flow field of a very low Reynolds cross-shaped jet was numerically investigated using the laminar model, and the large eddy simulation (LES) model. The corresponding results are compared with PIV and LDV measurements for a reference jet configuration. The objective is to assess the capability and limitations of numerical simulations in predicting the significant features of the cross-shaped jet flow when the flow is numerically resolved through the lobed diffuser. The laminar model was found to overestimate the global flow expansion and the jet volumetric flow rate. The laminar model fails also in reproducing the behavior of the flow velocity fields and of the axial velocity decay which are of capital importance in our research field. For the prediction of such integral quantities, LES is more appropriate. Given the computational limitations, it reproduces reasonably well the mean flow behavior in the ten first equivalent diameters from the orifice, where the mesh grid was very refined. The streamwise velocity distributions, the streamwise velocity decay and the volumetric flow rate predicted by the LES model are reasonably well reproduced from the perspective of the aimed application.

The temporal dynamics of the flow was not found to be completely in accord with the experimental data, but some similarities with the experimental results are rather intriguing and incite us on the one hand to search for an improvement in the numerical approach and on the other hand to deepen the experimental investigation for achieving a larger data base for the validation of the numerical results. This way, a volumetric PIV investigation of the low Reynolds jet flow is in preparation at the LaSIE laboratory.

## Data Availability

Specific sets of data are available on request.

## Nomenclature

D_e_	Equivalent diameter based on the exit area of the elementary orifice (mm)
D_TR_	Jet flow dimension (mm)
Q	Flow rate (m^3^/s)
Re	Reynolds number (-)
$U_{0c}$	Centerline jet velocity (m/s)
U_0mean_	Orifice’s mean exit velocity (m/s)
U:V,W	Velocity components in X, Y, Z direction (m/s)
X, Y, Z	Cartesian coordinates (m)
y^+^	Non-dimensional wall distance
$\tau_{YZ}$	Wall shear stress (Pa)
$\omega_{X}$	Streamwise vorticity component (1/s)
μ	Dynamic viscosity (Pa s)
